# From awareness to action: a comparative study of family medical history and preventive health behaviors in Saudi Arabia and Syria

**DOI:** 10.3389/fpubh.2026.1873367

**Published:** 2026-07-01

**Authors:** Bassel Tarakji, Adel M. Alqarni, Anas Alsalhani, Faisal Mehsen Alali, Nasser Raqe Alqhtani, Basem Sabbagh, Abdullah bin nabhan, Faisal S. Alhedyan, Yousef Alkhaibari, Abdullah Almansour, Rafif Alshenaiber, Abdullah Ibrahim Alenazi, Abdulaziz Mohammed Al Habshan, Mohammed Alshammari, Mana Alqahtani, Feras Baba, Azza Sioufi, Mohammad Zakaria Nassani

**Affiliations:** 1 Department of Oral Maxillofacial Surgery and Diagnostic Sciences, College of Dentistry, Prince Sattam Bin Abdulaziz University, Alkharj, Saudi Arabia; 2 Department of Oral Pathology, Faculty of Dentistry, University of Aleppo, Aleppo, Syria; 3 Department of Medical Laboratory, College of Applied Medical Sciences, Prince Sattam bin Abdulaziz University, Al-Kharj, Saudi Arabia; 4 Department of Dentistry, Vision Colleges, Riyadh, Saudi Arabia; 5 Department of Histology and Pathology, Faculty of Dentistry, University of Hama, Hama, Syria; 6 Department of Orthodontics, Faculty of Dentistry, Al-Wataniya Private University, Hama, Syria; 7 Department of Oral and Maxillofacial Surgery and Diagnostic Sciences, College of Dentistry, Prince Sattam Bin Abdulaziz University, Al Kharj, Saudi Arabia; 8 Department of Oral and Maxillofacial and Diagnostic Science, College of Dentistry, Prince Sattam Bin Abdulaziz University, Al-Kharj, Saudi Arabia; 9 Department of Prosthetic Dental Sciences, Prince Sattam bin Abdulaziz University, Al-Kharj, Saudi Arabia; 10 College of Dentistry, Prince Sattam Bin Abdulaziz University, Al-Kharj, Saudi Arabia; 11 Department of Surgery, Faculty of Medicine, University of Tabuk, Tabuk, Saudi Arabia; 12 Department of Orthodontics, Faculty of Dentistry, University of Aleppo, Aleppo, Syria; 13 Department of Obstetrics and Gynecology, Aljazeera Hospital, Riyadh, Saudi Arabia; 14 Department of Restorative and Prosthetic Dental Sciences, College of Dentistry, Dar Al Uloom University, Riyadh, Saudi Arabia; 15 Department of Removable Prosthodontics, Faculty of Dentistry, University of Aleppo, Aleppo, Syria

**Keywords:** awareness, family medical history, genetic diseases, preventive behavior, Saudi Arabia, Syria

## Abstract

**Background:**

Family medical history (FMH) is a practical and cost-effective tool for identifying individuals at increased risk of genetic and chronic diseases. However, its effective utilization depends on awareness, attitudes, and the translation of these factors into health-related practices and preventive behaviors, which may vary across populations.

**Objective:**

To assess and compare FMH awareness, attitudes, health-related practices, and preventive behaviors among adults in Saudi Arabia and Syria.

**Methods:**

A cross-sectional study was conducted using a structured online questionnaire distributed via digital platforms. A total of 586 participants (Saudi Arabia: 307; Syria: 279) were included. Four domains were evaluated: awareness, attitudes, health-related practices, and preventive behaviors. Outcomes were classified as favorable using Bloom’s cut-off (≥60%). Data were analyzed using descriptive statistics, chi-square tests, and multivariate logistic regression.

**Results:**

Favorable levels were observed in 53.4% for awareness, 74.2% for attitudes, 48.5% for health-related practices, and 44.0% for preventive behaviors. Although a majority of participants demonstrated awareness of FMH and positive attitudes toward genetic screening, engagement in preventive actions was limited. Only 45.2% had undergone medical testing and 45.1% reported lifestyle modifications based on familial risk. A pronounced intention–behavior gap was identified, with 74.2% expressing willingness to undergo screening but substantially fewer reporting actual engagement. Participants from Saudi Arabia exhibited significantly higher levels of preventive practices compared to those from Syria (*p* < 0.001). Multivariate analysis identified age, gender, education level, and country of residence as significant predictors of preventive behaviors.

**Conclusion:**

Despite moderate awareness and favorable attitudes, engagement in preventive practices remains suboptimal, indicating a clear gap between intention and action. These findings highlight the need for context-specific, multi-level strategies that address both behavioral and structural barriers to improve the effective use of FMH in preventive healthcare.

## Introduction

Genetic diseases and hereditary conditions represent a significant and growing public health concern worldwide, particularly in regions where consanguinity and familial aggregation of chronic diseases are common (1) In the Middle East, conditions such as hemoglobinopathies, cardiovascular diseases, diabetes, and certain cancers contribute substantially to long-term morbidity and healthcare burden (2). Family medical history (FMH) is widely recognized as a practical and cost-effective tool for identifying individuals at increased risk of these conditions and guiding early detection and preventive strategies. However, the effectiveness of FMH depends largely on individuals’ awareness, attitudes, and engagement in health-related practices, which may vary considerably across populations (3, 4).

In Saudi Arabia, national initiatives such as premarital screening programs and public health campaigns have been implemented to reduce the burden of genetic diseases. Despite these efforts, awareness and understanding of genetic risk and FMH remain variable, and do not consistently translate into appropriate health practices or preventive behaviors. Misconceptions regarding disease severity, susceptibility, and treatability may persist, limiting the effectiveness of these initiatives (3). In contrast, Syria faces additional challenges, including healthcare system disruption, limited access to preventive services, and reduced opportunities for public health education, which may further influence both awareness and engagement in preventive health behaviors (5).

Family medical history plays a central role in shaping individuals’ perceptions of disease susceptibility. Knowledge of affected relatives, recognition of inherited risk, and awareness of recommended screening measures can influence attitudes toward genetic diseases, including perceived seriousness, personal vulnerability, and willingness to adopt preventive actions such as medical testing or lifestyle modification. However, individuals may underestimate their familial risk or lack sufficient knowledge to make informed health decisions, resulting in inconsistencies between awareness, beliefs, and actual health behaviors (6, 7).

Importantly, the relationship between awareness, attitudes, and health behavior is complex and not always linear. While individuals may demonstrate positive attitudes toward genetic diseases and express willingness to engage in prevention, this does not necessarily result in corresponding health practices or participation in screening programs. Structural and contextual factors including access to healthcare services, availability of screening programs, and communication within families play a critical role in determining whether knowledge is translated into action. Barriers to collecting and discussing FMH, as well as limited integration of genetic risk assessment into routine healthcare, remain important challenges (8–10).

Given these considerations, it is essential to examine FMH awareness, attitudes, health-related practices, and preventive behaviors as interconnected domains rather than isolated constructs. Evaluating these domains in different country contexts can provide insight into how healthcare systems and sociocultural factors shape health behavior and identify potential gaps between knowledge and action.

This study aims to assess and compare family medical history awareness, attitudes toward genetic diseases, health-related practices, and preventive behaviors among adults in Saudi Arabia and Syria. Specifically, the study seeks to examine disparities between the two countries and to identify gaps between awareness, attitudes, and actual engagement in preventive behaviors. In addition, it aims to determine key demographic predictors such as age, gender, education level, and country of residence associated with these outcomes, in order to generate evidence that can inform targeted and context-specific public health strategies for improving early detection and prevention of genetic and chronic diseases.

## Materials and methods

### Study design, setting, and participants

This study employed a descriptive cross-sectional survey design to assess family medical history (FMH) awareness, attitudes toward genetic diseases, health-related practices, and preventive behaviors among adults residing in Saudi Arabia and Syria. Health-related practices were defined as family medical history (FMH)-related actions and intentions undertaken by participants, such as discussing family medical history with relatives, seeking information about familial diseases, advising others regarding screening, planning future screening, and making lifestyle modifications based on perceived familial risk. In contrast, preventive behaviors were defined as actual engagement in preventive healthcare activities, including undergoing blood pressure measurement, blood sugar testing, cholesterol testing, and complete blood count screening (11). The study aimed to compare these domains between the two countries and to identify factors associated with favorable awareness, attitudes, practices, and preventive behaviors.

Data were collected using a structured, self-administered online questionnaire developed and distributed via Google Forms. The survey was disseminated through multiple digital platforms, including WhatsApp, X (formerly Twitter), Facebook, and Telegram, to enhance population reach. The questionnaire consisted of five sections: (1) demographic characteristics, including family disease history; (2) awareness of family medical history; (3) attitudes toward genetic diseases; (4) health-related practices; and (5) preventive behaviors. All items were measured using predefined categorical (yes/no) response options.

Participation was voluntary, and electronic informed consent was obtained from all respondents prior to participation. Eligible participants were individuals aged ≥18 years residing in Saudi Arabia or Syria. The study was conducted in accordance with the Strengthening the Reporting of Observational Studies in Epidemiology (STROBE) guidelines (12) and adhered to the Checklist for Reporting Results of Internet E-Surveys (CHERRIES) (13). Ethical approval was obtained from the relevant institutional review board (SCBR, 369–2024), and all procedures were conducted in accordance with the Declaration of Helsinki. Participant anonymity and confidentiality were strictly maintained.

### Sample size calculation

The sample size was calculated using OpenEpi (Open Source Epidemiologic Statistics for Public Health, Version 3.01) (14). For the two-country comparison, the minimum required sample size was estimated separately for Saudi Arabia and Syria, assuming an expected prevalence of 50%, a 95% confidence level, and 7% absolute precision. A precision level of 7% was selected as a pragmatic compromise between statistical precision and feasibility of participant recruitment across the two study settings. In the absence of prior prevalence estimates for the combined study outcomes, a conservative prevalence of 50% was assumed to maximize the required sample size.

This yielded a minimum of 196 participants per country. The final sample included 586 participants, exceeding the required sample size for each country and overall, thereby ensuring adequate statistical power for comparative and regression analyses.

### Questionnaire development and validation

The questionnaire was initially developed in English based on a comprehensive review of literature on genetic diseases, family medical history, and preventive health behaviors (8, 15). It was structured according to the knowledge, attitude, and practice (KAP) framework (16) and adapted to assess four key domains: awareness of family medical history, attitudes toward genetic diseases, health-related practices, and preventive behaviors, consistent with the objectives of the study.

The questionnaire was translated into Arabic using a forward translation process, followed by expert review to ensure linguistic accuracy and conceptual equivalence, in line with established cross-cultural adaptation guidelines (17).

Content and face validity were established through evaluation by three experts in genetic diseases and public health. A pilot study involving 20 participants was conducted to assess clarity and feasibility. Feedback from the pilot phase was used to refine the questionnaire, and pilot responses were excluded from the final analysis (13).

### Data collection and management

Because the survey was distributed through multiple online platforms, the total number of individuals who viewed or accessed the survey link could not be determined; therefore, a conventional response rate could not be calculated. All questionnaire items were configured as mandatory in Google Forms, preventing submission of partially completed questionnaires. Consequently, no incomplete responses were recorded, and all submitted questionnaires were included in the final analysis.

Because the survey was anonymous, duplicate submissions could not be completely prevented. However, potential duplicate responses were assessed during data cleaning by reviewing response timestamps, response patterns, and demographic characteristics for evidence of repeated entries. No apparent duplicate responses were identified in the final dataset.

Following data collection, responses were exported, coded, and anonymized. The dataset was screened for completeness and consistency prior to statistical analysis.

### Operational definitions

Each domain was assessed using a set of binary (yes/no) items. The awareness domain comprised 6 items, the attitude domain 11 items, the practice domain 7 items, and the preventive behavior domain 4 items, consistent with the study instrument and analysis framework.

Domain scores were categorized using Bloom’s cut-off point (≥60%) (18, 19). Participants who answered “yes” to ≥60% of items within a domain were classified as having favorable outcomes, while those scoring below this threshold were classified as unfavorable.

Accordingly, participants scoring ≥4 out of 6 were categorized as having favorable awareness, ≥7 out of 11 as having favorable attitude, ≥5 out of 7 as having favorable practice, and ≥3 out of 4 as having favorable preventive behavior.

Comorbidity was assessed as a self-reported variable and defined as the presence of one or more chronic medical conditions (e.g., hypertension, diabetes, cardiovascular disease, or other long-term illnesses) at the time of the survey.

### Reliability analysis

Internal consistency reliability was assessed using Cronbach’s alpha coefficient (20) in IBM SPSS Statistics version 25. Reliability analysis was conducted on item-level responses within each domain, with reverse coding applied where necessary.

The overall questionnaire demonstrated good internal consistency (*α* = 0.86). Domain-specific Cronbach’s alpha values were 0.69 for awareness, 0.58 for attitude, 0.75 for practice, and 0.76 for preventive behavior. Values ≥0.70 were considered acceptable, while slightly lower values were deemed acceptable in exploratory research (21).

### Statistical analysis

Data were analyzed using the Statistical Package for the Social Sciences (SPSS), version 25.0 (IBM Corp., Armonk, NY, USA). Descriptive statistics were used to summarize participant characteristics and responses across the family medical history domains. Categorical variables were presented as frequencies and percentages.

Comparisons between participants from Saudi Arabia and Syria across family medical history domains were performed using the chi-square (χ^2^) test.

For the main outcome measures, participants’ responses were aggregated and classified as favorable or unfavorable for awareness, attitude, practice, and preventive behavior based on Bloom’s cut-off point (≥60%) (18, 22). Specifically, participants were categorized as having favorable levels if they provided affirmative responses to at least 60% of the items within each domain (i.e., ≥4/6 for awareness, ≥7/11 for attitude, ≥5/7 for practice, and ≥3/4 for preventive behavior), and unfavorable otherwise. Bar charts and a heatmap were generated to visually present the distribution of participants with favorable and unfavorable levels across the four domains of family medical history, and the proportion of participants with favorable levels in each domain, with color intensity reflecting percentage values.

Bivariate analysis using chi-square tests was conducted to assess associations between demographic variables and favorable outcomes across the four domains of family medical history.

To account for multiple comparisons, Bonferroni correction was applied. For item-level comparisons (34 tests), a significance threshold of *p* < 0.0015 (0.05/34) was used. For associations between demographic variables and outcomes (20 tests), a threshold of *p* < 0.0025 (0.05/20) was applied.

Multivariate binary logistic regression analysis was performed to identify independent predictors of favorable awareness, attitude, practice, and preventive behavior. Variables included in the models were age, gender, education level, comorbidity status, and country. Adjusted odds ratios (ORs) with 95% confidence intervals (CIs) were reported.

Model diagnostics were conducted to evaluate the adequacy of the regression models. Goodness-of-fit was assessed using the Hosmer–Lemeshow test, with *p* > 0.05 indicating an acceptable fit. Multicollinearity among independent variables was assessed using variance inflation factors (VIFs), with values <5 considered acceptable.

All statistical tests were two-tailed, and a *p*-value <0.05 was considered statistically significant unless otherwise adjusted for multiple comparisons.

### Bias mitigation

Several methodological strategies were implemented to minimize potential sources of bias throughout the study. To reduce selection bias, the survey was disseminated across multiple digital platforms and community networks to enhance the diversity and representativeness of participants from both Saudi Arabia and Syria. Participation was entirely voluntary, and no incentives were provided, thereby reducing the likelihood of coerced responses.

To address information bias, a structured questionnaire with standardized closed-ended (yes/no) items was employed to ensure consistency in data collection. The instrument underwent expert review and pilot testing to improve clarity, relevance, and comprehensibility, thereby minimizing the risk of misinterpretation. The design and reporting of the online survey adhered to the CHERRIES (Checklist for Reporting Results of Internet E-Surveys) guidelines (13) ensuring methodological rigor in web-based data collection.

Recall bias was minimized by focusing on participants’ current knowledge, perceptions, and recent health-related behaviors rather than distant past events. In addition, strict anonymity and confidentiality were maintained, which likely reduced social desirability bias and encouraged accurate self-reporting.

Furthermore, the study design and reporting followed the STROBE (Strengthening the Reporting of Observational Studies in Epidemiology) guidelines (12) enhancing transparency and methodological quality. Data quality was maintained through systematic screening procedures, including the exclusion of responses from individuals who did not provide informed consent. Potential duplicate entries were reviewed based on response patterns and timestamps, and no duplicates were identified. As all questionnaire items were mandatory, no incomplete responses were recorded. Only responses from eligible participants who approved participation were included in the final analysis (*n* = 586), thereby strengthening the validity and reliability of the study findings.

## Results

### Demographic characteristics of participants

The demographic characteristics of participants are summarized in Table 1. A total of 586 participants were included, with 307 (52.4%) from Saudi Arabia and 279 (47.6%) from Syria. Over half were aged ≤30 years (52.4%), while only 9.4% were >50 years. The Syrian sample was predominantly younger, whereas most Saudi participants were aged 31–50 years (58.0%).

**Table 1:** Demographic characteristics of participants by country.

Demographics	Sample 586 (100%)	Saudi Arabia 307 (52.4%)	Syria 279 (47.6%)
Age (years)			
≤30	307 (52.4%)	93 (30.3%)	214 (76.7%)
31–50	224 (38.2%)	178 (58.0%)	46 (16.5%)
>50	55 (9.4%)	36 (11.7%)	19 (6.8%)
Gender			
Male	388 (66.2%)	254 (82.7%)	134 (48.0%)
Female	198 (33.8%)	53 (17.3%)	145 (52.0%)
Education Level			
Intermediate school or less	67 (11.4%)	57 (18.5%)	10 (3.6%)
Secondary school	108 (18.4%)	86 (28.0%)	22 (7.9%)
University/College	411 (70.1%)	164 (53.4%)	247 (88.5%)
Comorbidity			
No	502 (85.7%)	243 (79.2%)	259 (92.8%)
Yes	84 (14.3%)	64 (20.8%)	20 (7.2%)
Family disease history			
Family history of hypertension	347 (59.2%)	226 (73.6%)	121 (43.4%)
Family history of diabetes	250 (42.7%)	154 (50.2%)	96 (34.4%)
Family history of cancer	233 (39.8%)	198 (64.5%)	35 (12.5%)
Family history of heart disease	234 (39.9%)	160 (52.1%)	74 (26.5%)
Family history of Alzheimer’s disease	182 (31.1%)	173 (56.4%)	9 (3.2%)
Genetic diseases in immediate family	316 (53.9%)	137 (44.6%)	179 (64.2%)

Males comprised 66.2% of the sample, with a higher proportion in Saudi Arabia (82.7%), while females slightly predominated in Syria (52.0%). Most participants had a university-level education (70.1%), particularly in Syria (88.5%), whereas lower education levels were more common in Saudi Arabia (18.5%).

Overall, 14.3% reported at least one chronic disease, more frequently in Saudi Arabia (20.8%) than Syria (7.2%). Hypertension was the most commonly reported family condition (59.2%), followed by diabetes (42.7%), cancer (39.8%), and heart disease (39.9%), with generally higher prevalence in the Saudi sample. Alzheimer’s disease was reported by 31.1%.

More than half of participants (53.9%) reported genetic diseases in their immediate family, with a higher proportion in Syria (64.2%) than in Saudi Arabia (44.6%).

### Family medical history awareness, attitudes, health practices, and preventive behavior

Table 2 presents participants’ awareness of family medical history and related factors. Overall, 60.9% reported being aware of their family medical history, with a significantly higher proportion in the Syrian sample compared to Saudi Arabia (75.3% vs. 47.9%, *p* < 0.001).

**Table 2:** Family medical history awareness, attitudes, health practices, and preventive behavior among participants based on country.

Domain and survey items	% of “yes” answers by country			*p*-value
	Sample 586 (100%)	Saudi Arabia 307 (52.4%)	Syria 279 (47.6%)	
1. Awareness of family medical history				
1.1 Do you know your family medical history?	357 (60.9%)	147 (47.9%)	210 (75.3%)	<0.001*
1.2 Are you aware of the possibility of genetic diseases in your family?	279 (47.6%)	187 (60.9%)	92 (33.0%)	<0.001*
1.3 Do you know family members who have genetic diseases?	343 (58.5%)	208 (67.8%)	135 (48.4%)	<0.001*
1.4 Do you know lifestyle changes that can improve your health regarding these diseases?	363 (61.9%)	207 (67.4%)	156 (55.9%)	0.005
1.5 Do you know the possible medical complications related to your family medical history?	351 (59.9%)	188 (61.2%)	163 (58.4%)	0.542
1.6 Do you know the early screening methods recommended based on your family medical history?	337 (57.5%)	190 (61.9%)	147 (52.7%)	0.030
2. Attitude (Beliefs) toward family medical history and genetic diseases				
2.1 I am interested in my family medical history	428 (73.0%)	196 (63.8%)	232 (83.2%)	<0.001*
2.2 It is difficult to know diseases in my family history	350 (59.7%)	244 (79.5%)	106 (38.0%)	<0.001*
2.3 I have thought about how my family history affects my health	386 (65.9%)	191 (62.2%)	195 (69.9%)	0.061
2.4 I would change my lifestyle to prevent early symptoms of genetic disease	502 (85.7%)	266 (86.6%)	236 (84.6%)	0.554
2.5 I am willing to undergo expensive or uncomfortable tests to live longer	446 (76.1%)	238 (77.5%)	208 (74.6%)	0.456
2.6 Having a family member with a genetic disease increases my risk	442 (75.4%)	226 (73.6%)	216 (77.4%)	0.331
2.7 Genetic diseases may be fatal	402 (68.6%)	210 (68.4%)	192 (68.8%)	0.985
2.8 Everyone should undergo genetic screening regardless of family history	473 (80.7%)	248 (80.8%)	225 (80.6%)	1.000
2.9 I believe disease risk increases if a family member is affected	420 (71.7%)	207 (67.4%)	213 (76.3%)	0.021
2.10 I believe knowledge of genetic diseases improves quality of life	459 (78.3%)	232 (75.6%)	227 (81.4%)	0.110
2.11 I believe genetic diseases are treatable	353 (60.2%)	220 (71.7%)	133 (47.7%)	<0.001*
3. Practice related to family medical history				
3.1 Have you undergone medical tests because of your knowledge of your family medical history?	265 (45.2%)	198 (64.5%)	67 (24.0%)	<0.001*
3.2 Have you made lifestyle changes because of your knowledge of your family medical history?	264 (45.1%)	156 (50.8%)	108 (38.7%)	0.004
3.3 Do you plan to undergo specific screenings in the future when reaching the recommended age?	435 (74.2%)	235 (76.5%)	200 (71.7%)	0.211
3.4 Have you searched or asked about your family medical history?	380 (64.8%)	218 (71.0%)	162 (58.1%)	0.001*
3.5 Have you discussed your family medical history and its importance with your family?	351 (59.9%)	193 (62.9%)	158 (56.6%)	0.146
3.6 Do you warn others about genetic diseases?	391 (66.7%)	184 (59.9%)	207 (74.2%)	<0.001*
3.7 Have you ever advised others to undergo screening for genetic diseases?	380 (64.8%)	230 (74.9%)	150 (53.8%)	<0.001*
4. Preventive behavior				
4.1 Have you had your blood pressure checked during the past year?	363 (61.9%)	199 (64.8%)	164 (58.8%)	0.156
4.2 Have you had your blood sugar tested during the past year?	298 (50.9%)	183 (59.6%)	115 (41.2%)	<0.001*
4.3 Have you had your cholesterol level tested during the past year?	256 (43.7%)	190 (61.9%)	66 (23.7%)	<0.001*
4.4 Have you had a complete blood count (CBC) test during the past year?	309 (52.7%)	181 (59.0%)	128 (45.9%)	0.002

*Denotes a significant difference at *p* < 0.0015, as indicated by χ^2^ statistics with Bonferroni adjustment (0.05/34).

In contrast, a higher proportion of participants in Saudi Arabia reported awareness of the possibility of genetic diseases in their family (60.9% vs. 33.0%, *p* < 0.001) and knowledge of affected family members (67.8% vs. 48.4%, *p* < 0.001). Furthermore, 59.9% of participants reported awareness of potential medical complications related to family medical history (61.2% in Saudi Arabia vs. 58.4% in Syria, *p* = 0.542), while 57.5% reported knowledge of recommended early screening methods (61.9% vs. 52.7%, *p* = 0.030).

Overall, attitudes toward family medical history and genetic diseases were generally positive. A majority of participants expressed interest in their family medical history (73.0%), and 80.7% believed that everyone should undergo genetic screening regardless of family history. Additionally, 71.7% believed that disease risk increases if a family member is affected, and 78.3% agreed that knowledge of genetic diseases improves quality of life. However, 59.7% reported that it is difficult to obtain information about diseases within their family.

Significant differences between Saudi Arabia and Syria were observed in several items. Participants from Syria were more likely to report interest in their family medical history (83.2% vs. 63.8%, *p* < 0.001), whereas those from Saudi Arabia more frequently reported difficulty in obtaining family health information (79.5% vs. 38.0%, *p* < 0.001). In addition, a higher proportion of participants in Saudi Arabia believed that genetic diseases are treatable (71.7% vs. 47.7%, *p* < 0.001).

In terms of practice, several favorable behaviors were observed. A majority of participants reported planning to undergo recommended screenings in the future (74.2%). Additionally, 64.8% had searched for or asked about their family medical history (71.0% in Saudi Arabia vs. 58.1% in Syria, *p* = 0.001), 66.7% had warned others about genetic diseases (59.9% vs. 74.2%, *p* < 0.001), and 64.8% had advised others to undergo screening (74.9% vs. 53.8%, *p* < 0.001). Furthermore, 59.9% reported discussing their family medical history with relatives (62.9% vs. 56.6%, *p* = 0.146).

Less common practices included undergoing medical tests due to family history (45.2%; 64.5% in Saudi Arabia vs. 24.0% in Syria, *p* < 0.001) and making lifestyle changes based on such knowledge (45.1%; 50.8% vs. 38.7%, *p* = 0.004).

As regards preventive behavior, the results were suboptimal. Only 43.7% of participants reported having their cholesterol level tested during the past year (61.9% in Saudi Arabia vs. 23.7% in Syria, *p* < 0.001). Similarly, 50.9% had their blood sugar tested (59.6% vs. 41.2%, *p* < 0.001), and 52.7% had undergone a complete blood count (CBC) test (59.0% vs. 45.9%, *p* = 0.002). Blood pressure measurement was the most commonly reported behavior, with 61.9% having it checked during the past year (64.8% vs. 58.8%, *p* = 0.156).

### Levels of awareness, attitude, practice, and preventive behavior regarding family medical history

**Figure 1:**
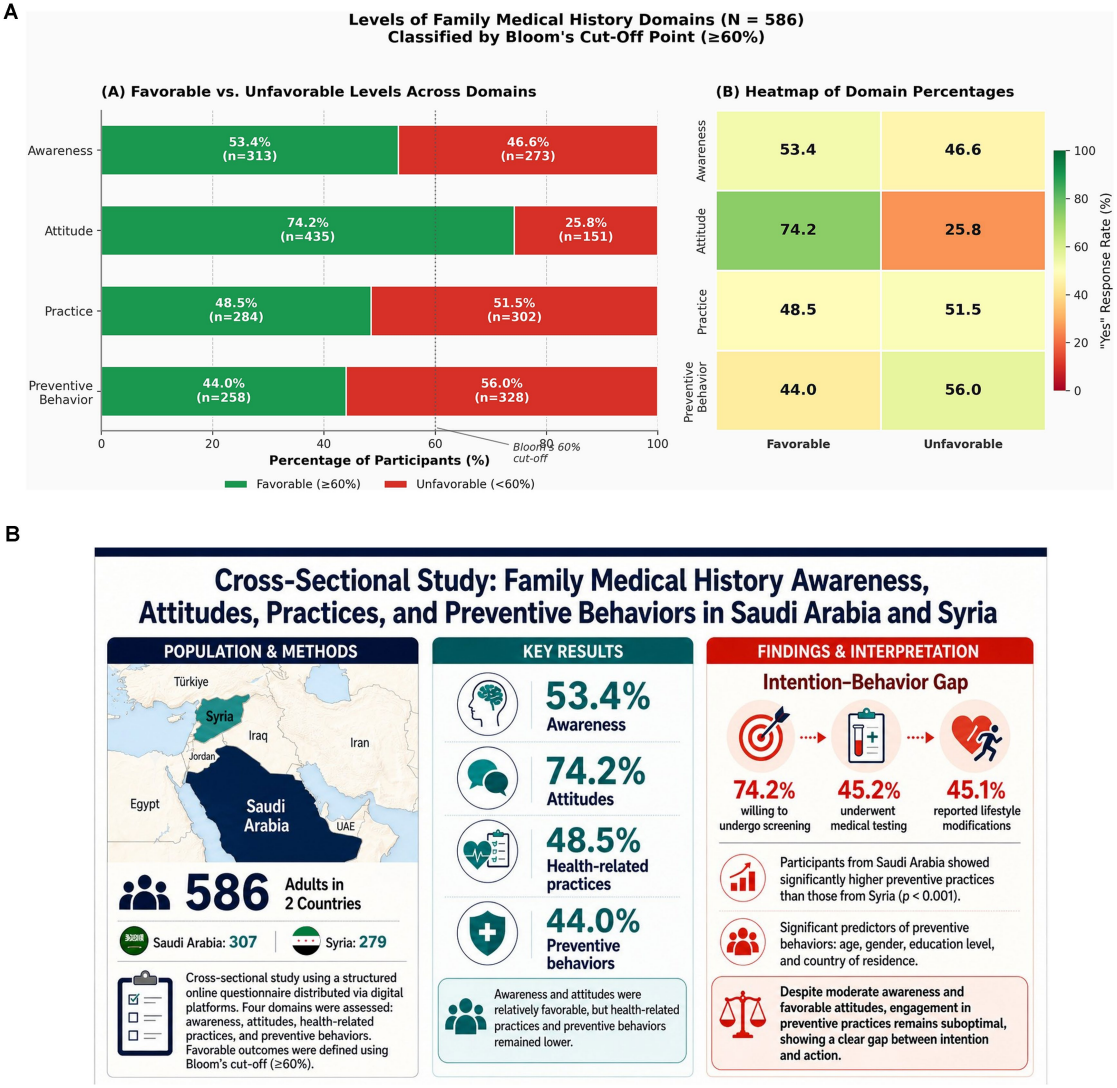
**(A)** Distribution of participants with favorable and unfavorable levels across the four domains of family medical history (awareness, attitudes, practices, and preventive behaviors), classified using Bloom’s cut-off point (≥60%). **(B)** Heatmap showing the proportion of participants with favorable levels across the four domains of family medical history, with color intensity representing the percentage of participants in each domain (n = 586).

Participants were classified as having favorable awareness, attitude, practice, and preventive behavior if they answered “yes” to ≥60% of items within each domain, based on Bloom’s cut-off point. The analysis showed that 53.4% of participants had favorable awareness, 74.2% had favorable attitudes, 48.5% demonstrated favorable practices, and 44.0% exhibited favorable preventive behavior (Table 3; Figure 1). When stratified by country, favorable awareness was observed among 56.4% of participants from Saudi Arabia and 50.2% from Syria (*p* = 0.158). Favorable attitudes were reported by 69.7% of participants from Saudi Arabia and 79.2% from Syria (*p* = 0.011). Favorable practices were observed in 53.4 and 43.0% of participants from Saudi Arabia and Syria, respectively (*p* = 0.015). The largest difference was observed for preventive behavior, with favorable levels reported by 56.0% of participants from Saudi Arabia compared with 30.8% from Syria (*p* < 0.001; Table 4).

**Table 3:** Levels of awareness, attitude, practice, and preventive behavior regarding family medical history among participants classified by Bloom’s cut-off point (≥60%).

Domain	Items	Cut-off	Favorable n (%)	Unfavorable n (%)
Awareness of family medical history	6	≥60% (≥4/6)	313 (53.4%)	273 (46.6%)
Attitude (Beliefs) toward family medical history and genetic diseases	11	≥60% (≥7/11)	435 (74.2%)	151 (25.8%)
Practice related to family medical history	7	≥60% (≥5/7)	284 (48.5%)	302 (51.5%)
Preventive behavior	4	≥60% (≥3/4)	258 (44.0%)	328 (56.0%)

Participants were classified as having favorable awareness, attitude, practice, and preventive behavior if they answered “yes” to ≥60% of the listed questions, or unfavorable if they answered “no” (scored <60%) (Bloom’s cut-off point).

**Table 4:** Association between demographics and favorable levels of family medical history awareness, attitudes, practices, and preventive behaviors.

Demographics	Comparison Outcomes: Favorable Awareness, Attitudes, Practices, and Preventive Behaviors							
	Awareness	*p*-value	Attitude	*p*-value	Practice	*p*-value	Preventive behavior	*p*-value
Sample	313 (53.4%)	**-**	435 (74.2%)	**-**	284 (48.5%)	**-**	258 (44.0%)	**-**
Age (years)								
≤30	153 (49.8%)	0.007	247 (80.5%)	<0.001*	137 (44.6%)	0.143	98 (31.9%)	<0.001*
31–50	137 (61.2%)		154 (68.8%)		119 (53.1%)		126 (56.2%)	
>50	23 (41.8%)		34 (61.8%)		28 (50.9%)		34 (61.8%)	
Gender								
Male	213 (54.9%)	0.357	277 (71.4%)	0.036	207 (53.4%)	0.001*	202 (52.1%)	<0.001*
Female	100 (50.5%)		158 (79.8%)		77 (38.9%)		56 (28.3%)	
Education level								
Intermediate school or less	47 (70.1%)	0.003	44 (65.7%)	0.097	41 (61.2%)	0.053	42 (62.7%)	0.005
Secondary school	47 (43.5%)		76 (70.4%)		46 (42.6%)		44 (40.7%)	
University/College	219 (53.3%)		315 (76.6%)		197 (47.9%)		172 (41.8%)	
Comorbidity								
No	263 (52.4%)	0.274	378 (75.3%)	0.191	238 (47.4%)	0.259	210 (41.8%)	0.013
Yes	50 (59.5%)		57 (67.9%)		46 (54.8%)		48 (57.1%)	
Country								
Saudi Arabia	173 (56.4%)	0.158	214 (69.7%)	0.011	164 (53.4%)	0.015	172 (56.0%)	<0.001*
Syria	140 (50.2%)		221 (79.2%)		120 (43.0%)		86 (30.8%)	

*Denotes a significant difference at *p* < 0.0025, as indicated by χ^2^ statistics with Bonferroni adjustment (0.05/20).

### Association between demographics and favorable levels of family medical history domains

Chi-square analysis with Bonferroni adjustment showed that age was significantly associated with attitude (*p* < 0.001) and preventive behavior (*p* < 0.001). Younger participants (≤30 years) demonstrated higher levels of favorable attitude (80.5%), compared to those aged 31–50 years (68.8%) and those >50 years (61.8%). In contrast, favorable preventive behavior increased with age, from 31.9% among those ≤30 years to 56.2% in those aged 31–50 years and 61.8% among those >50 years.

Gender was significantly associated with practice (*p* = 0.001) and preventive behavior (*p* < 0.001), with males demonstrating higher levels of favorable practice (53.4% vs. 38.9%) and preventive behavior (52.1% vs. 28.3%) compared to females.

Country of residence was significantly associated with preventive behavior (*p* < 0.001), with participants from Saudi Arabia showing higher favorable levels compared to those from Syria (56.0% vs. 30.8%).

No significant associations were observed between education level or comorbidity and the studied domains after Bonferroni adjustment (Table 4).

### Predictors associated with favorable levels of family medical history awareness, attitudes, practices, and preventive behaviors

Prior to interpretation, model diagnostics confirmed acceptable model performance. Hosmer–Lemeshow goodness-of-fit tests indicated adequate fit for all logistic regression models (all *p* > 0.05), and no evidence of problematic multicollinearity was detected among the independent variables (all VIFs < 5). Multivariate binary logistic regression analysis identified several significant predictors across the four outcome domains (Table 5).

**Table 5:** Predictors associated with favorable levels of family medical history awareness, attitudes, practices, and preventive behaviors.

Predictors/associated factors	Favorable awareness		Favorable attitude		Favorable practice		Favorable preventive behavior	
	OR (95% CI)	*p*	OR (95% CI)	*p*	OR (95% CI)	*p*	OR (95% CI)	*p*
Age (years)								
≤30	[Reference]		[Reference]		[Reference]		[Reference]	
31–50	1.44 (0.96–2.17)	0.076	0.63 (0.40–1.00)	0.050	1.07 (0.72–1.61)	0.729	1.73 (1.15–2.62)	0.009*
>50	0.60 (0.32–1.13)	0.114	0.47 (0.24–0.91)	0.024*	0.94 (0.51–1.74)	0.837	2.21 (1.16–4.21)	0.015*
Gender								
Male	[Reference]		[Reference]		[Reference]		[Reference]	
Female	0.97 (0.66–1.42)	0.874	1.18 (0.75–1.87)	0.473	0.64 (0.43–0.94)	0.022*	0.58 (0.38–0.87)	0.008*
Education level								
Intermediate school or less	[Reference]		[Reference]		[Reference]		[Reference]	
Secondary school	0.32 (0.16–0.61)	<0.001*	1.13 (0.59–2.20)	0.708	0.50 (0.27–0.94)	0.031*	0.46 (0.24–0.88)	0.019*
University/College	0.54 (0.30–0.98)	0.043*	1.32 (0.73–2.37)	0.360	0.75 (0.43–1.31)	0.305	0.78 (0.44–1.39)	0.398
Comorbidity								
No	[Reference]		[Reference]		[Reference]		[Reference]	
Yes	1.40 (0.84–2.33)	0.191	0.92 (0.54–1.57)	0.772	1.22 (0.75–2.00)	0.422	1.30 (0.78–2.16)	0.320
Country								
Saudi Arabia	[Reference]		[Reference]		[Reference]		[Reference]	
Syria	0.91 (0.60–1.37)	0.648	1.15 (0.72–1.84)	0.562	0.78 (0.52–1.17)	0.224	0.51 (0.34–0.78)	0.002*

OR, Odds ratio; CI, Confidence interval. The odds ratio and 95% confidence interval were calculated by a multivariate binary logistic model adjusted for age, gender, education level, and comorbidity. *Denotes significant difference at *p* < 0.05.

Favorable awareness of family medical history was significantly associated with education level. Participants with secondary education (OR = 0.32, 95% CI: 0.16–0.61, *p* < 0.001) and those with university/college education (OR = 0.54, 95% CI: 0.30–0.98, *p* = 0.043) were less likely to report favorable awareness compared to those with intermediate education or less.

Regarding attitudes, age was a significant predictor. Participants aged >50 years were less likely to report favorable attitudes compared with those aged ≤30 years (OR = 0.47, 95% CI: 0.24–0.91, *p* = 0.024).

For practice, gender and education level were significant predictors. Females were less likely than males to report favorable practices (OR = 0.64, 95% CI: 0.43–0.94, *p* = 0.022), and participants with secondary education were less likely to report favorable practices compared to those with lower education (OR = 0.50, 95% CI: 0.27–0.94, *p* = 0.031).

In terms of preventive behavior, age, gender, education level, and country were significant predictors. Participants aged 31–50 years (OR = 1.73, 95% CI: 1.15–2.62, *p* = 0.009) and those aged >50 years (OR = 2.21, 95% CI: 1.16–4.21, *p* = 0.015) were more likely to report favorable preventive behaviors compared to those aged ≤30 years. Females were less likely than males to report favorable behaviors (OR = 0.58, 95% CI: 0.38–0.87, *p* = 0.008). Additionally, participants with secondary education were less likely to report favorable preventive behaviors compared to those with intermediate education or less (OR = 0.46, 95% CI: 0.24–0.88, *p* = 0.019), and participants from Syria were less likely than those from Saudi Arabia (OR = 0.51, 95% CI: 0.34–0.78, *p* = 0.002).

## Discussion

### Principal findings and study contribution

This study provides a comprehensive and methodologically robust evaluation of family medical history (FMH) awareness, attitudes, health-related practices, and preventive behaviors within a comparative two-country context. In contrast to many previous studies that have examined these domains separately, the present study adopts an integrated framework that captures the progression from awareness and attitudes to health-related practices and preventive behaviors. This approach enables a more comprehensive understanding of how individuals perceive and respond to familial and genetic health risks. Such an integrated perspective is supported by recent evidence highlighting the importance of family history in risk stratification and the development of preventive health strategies (8, 23).

In addition, the inclusion of participants from two countries with distinct healthcare systems enhances the contextual relevance and external validity of the findings, particularly in settings with differing levels of access to preventive services. The use of a structured KAP-based questionnaire and standardized data collection procedures further strengthens the internal validity and reliability of the study. Moreover, the relatively large sample size contributes to improved statistical power and supports the generalizability of the results. Overall, these methodological strengths are consistent with current recommendations for population-based and behavioral health research in genomics and prevention (24, 25).

### Family medical history awareness and attitudinal perspectives

Awareness of family medical history was generally moderate, suggesting that many individuals remain insufficiently informed about familial disease risk and the preventive value of family health information (Table 3). At the item level, 60.9% of participants reported knowing their family medical history, while 57.5% were aware of recommended screening methods related to familial risk (Table 2). These findings are consistent with recent nationally representative data from adult populations in the United States, indicating that more than 60% of adults are aware of the health history of their biological relatives (26). Nevertheless, despite this level of awareness, important gaps persist in understanding the clinical relevance and preventive value of such information. For example, although most individuals acknowledge the importance of family health history, only a smaller proportion actively collect or document this information (26).

In contrast, attitudes toward family medical history and genetic diseases were largely positive, with 74.2% of participants demonstrating favorable attitudes (Table 3). A considerable proportion expressed interest in their family medical history (73.0%) and recognized the importance of genetic screening (80.7%; Table 2). These findings are consistent with previous studies conducted in Saudi Arabia and other populations, which report generally favorable perceptions toward genetic testing and screening programs (27, 28).

Despite these positive attitudes, a substantial proportion of participants (59.7%) reported difficulty in obtaining information about diseases within their families (Table 2). This challenge aligns with population-based evidence indicating that many individuals encounter barriers when collecting family health history information (26). Such barriers may include limited communication among family members, uncertainty regarding the type of information required, and broader challenges such as limited health literacy, language differences, and restricted access to genetic services (29). These factors may impede the effective utilization of family medical history in preventive healthcare.

Overall, the findings suggest that although attitudes toward family medical history are generally favorable, awareness remains only moderate, and both structural and informational barriers may limit its effective application in disease prevention.

### Health-related practices and preventive behaviors

The Intention–Behavior Gap. Despite relatively favorable awareness and attitudes, participants demonstrated lower levels of engagement in health-related practices and preventive behaviors. Despite generally positive attitudes toward family medical history and disease prevention, actual engagement in preventive practices remained limited, indicating that favorable perceptions alone may be insufficient to drive preventive action (Table 3), suggesting a discrepancy between participants’ favorable attitudes and intentions toward prevention and their actual engagement in preventive practices. Similar discrepancies between awareness and preventive health behaviors have been documented in population-based studies conducted in high-income countries, including the United States and several European nations (30).

At the item level, engagement in key preventive measures remained modest. Only 45.2% of participants had undergone medical testing based on their family medical history, and a comparable proportion (45.1%) reported making lifestyle changes in response to perceived familial risk (Table 2). In contrast, a substantially higher proportion (74.2%) expressed intentions to undergo screening in the future. This discrepancy underscores the well-established intention–behavior gap, where expressed intentions do not consistently translate into actual preventive actions (31).

Preventive behaviors were inconsistently adopted across routine screening activities. Less than half of participants reported undergoing cholesterol testing (43.7%), while approximately half reported blood sugar testing (50.9%) and complete blood count screening (52.7%) within the past year (Table 2). Blood pressure monitoring was the most commonly reported behavior (61.9%), indicating moderate but not universal engagement. These findings are consistent with evidence demonstrating suboptimal uptake of preventive screening services despite awareness of their importance (32).

Several factors may contribute to this gap, including limited access to healthcare services and behavioral determinants such as perceived susceptibility and perceived barriers to action. These constructs are central components of established health behavior frameworks used to explain preventive health practices (33).

Overall, these findings suggest that while individuals demonstrate awareness and positive attitudes toward family medical history, this does not consistently translate into sustained engagement in health-related practices and preventive behaviors. Addressing this gap requires targeted strategies that enhance access to screening services and support the translation of health intentions into preventive action.

### Country-level disparities in family medical history utilization

Notable differences were observed between participants from Saudi Arabia and Syria across multiple domains, including awareness, attitudes, health-related practices, and preventive behaviors. These variations underscore the influence of healthcare system structure, accessibility, and sociocultural context on health-related behaviors, as consistently reported in studies examining disparities in preventive healthcare utilization (34, 35).

In terms of awareness, participants from Syria were more likely to report knowing their family medical history compared to those from Saudi Arabia (75.3% vs. 47.9%, *p* < 0.001). However, participants in Saudi Arabia demonstrated greater depth of awareness, including higher recognition of the possibility of genetic diseases within their families (60.9% vs. 33.0%, *p* < 0.001) and greater awareness of affected relatives (67.8% vs. 48.4%, *p* < 0.001). Knowledge of recommended screening methods was also higher among Saudi participants (61.9% vs. 52.7%, *p* = 0.030), suggesting that while awareness may be more widespread in Syria, it may be more clinically oriented in Saudi Arabia. Similar differences in the quality and applicability of health knowledge have been linked to variations in health education and engagement with healthcare systems (36).

Attitudes toward family medical history and genetic diseases were generally favorable in both countries, although distinct patterns were observed. Participants from Syria expressed greater interest in their family medical history (83.2% vs. 63.8%, *p* < 0.001), whereas those from Saudi Arabia were more likely to report difficulty obtaining family health information (79.5% vs. 38.0%, *p* < 0.001). In addition, belief in the treatability of genetic diseases was higher among Saudi participants (71.7% vs. 47.7%, *p* < 0.001). These differences may reflect variations in health literacy, perceptions of disease, and engagement with healthcare services (37).

More pronounced disparities were evident in health-related practices. Participants in Saudi Arabia demonstrated higher engagement in several key behaviors, including undergoing medical testing due to family history (64.5% vs. 24.0%, *p* < 0.001), adopting lifestyle modifications (50.8% vs. 38.7%, *p* = 0.004), and actively seeking information about family medical history (71.0% vs. 58.1%, *p* = 0.001). In contrast, participants from Syria were more likely to report warning others about genetic diseases (74.2% vs. 59.9%, *p* < 0.001), suggesting differences in behavioral expression and social communication patterns.

Substantial differences were also observed in preventive behaviors. Participants from Saudi Arabia consistently reported higher uptake of routine screening practices, including cholesterol testing (61.9% vs. 23.7%, *p* < 0.001), blood sugar testing (59.6% vs. 41.2%, *p* < 0.001), and complete blood count testing (59.0% vs. 45.9%, *p* = 0.002). Although blood pressure monitoring was also more common in Saudi Arabia, the difference was not statistically significant (64.8% vs. 58.8%, *p* = 0.156). These findings align with existing evidence demonstrating that disparities in healthcare access and screening infrastructure significantly influence the uptake of preventive services (38, 39).

These patterns are further supported by multivariate analysis, which identified country of residence as a significant predictor of preventive behavior. Participants from Syria were significantly less likely to demonstrate favorable preventive behaviors compared to those from Saudi Arabia (OR = 0.51, 95% CI: 0.34–0.78, *p* = 0.002). This finding reinforces previous research highlighting the critical role of structural factors such as healthcare accessibility, resource availability, and system-level capacity in shaping preventive health behaviors (34, 36).

Overall, the results reveal clear country-level disparities. Participants in Saudi Arabia demonstrated greater engagement in health-related practices and preventive behaviors, whereas participants in Syria exhibited higher levels of basic awareness and interest in family medical history. These findings emphasize the importance of context-specific strategies that address structural barriers, enhance access to preventive services, and promote equitable utilization of healthcare resources across populations (37, 38). The relatively low uptake of preventive practices observed in the present study is consistent with findings from previous research in Saudi Arabia showing that awareness of health risks does not always translate into participation in preventive screening programs. Factors such as limited perceived susceptibility, time constraints, concerns regarding screening procedures, and inadequate physician recommendations have been reported as barriers to preventive healthcare utilization. In Syria, engagement in preventive healthcare may be further influenced by healthcare system disruptions, reduced accessibility of preventive services, financial constraints, and competing health priorities associated with prolonged socioeconomic challenges. These contextual factors may help explain the lower levels of preventive behavior observed despite generally favorable attitudes toward disease prevention (36–37–44).

### Determinants of awareness, practices, and preventive behaviors

Multivariate analysis identified several key determinants influencing family medical history–related outcomes, reflecting the multifactorial nature of health behavior. Age emerged as a significant predictor, with older individuals more likely to engage in preventive behaviors. This pattern may be attributed to increased health awareness, greater interaction with healthcare services, and a higher perceived susceptibility to disease with advancing age, as consistently reported in preventive health research (40).

Gender differences were also evident, with females demonstrating lower engagement in health-related practices and preventive behaviors. Such disparities have been observed in previous studies and may be influenced by sociocultural roles, differences in healthcare utilization, and competing responsibilities that can limit participation in preventive care (41).

Education level showed inconsistent associations across the study domains. Contrary to expectations, higher levels of formal education were not consistently associated with improved awareness or engagement in preventive behaviors. This finding suggests that general education alone may not be sufficient to promote effective health behavior change, highlighting the importance of health-specific literacy and targeted educational interventions (42). Further research, particularly qualitative in nature, is warranted to better understand the underlying reasons for this finding.

Notably, comorbidity was not significantly associated with any of the assessed outcomes. This indicates that the presence of chronic conditions does not necessarily translate into increased awareness or engagement in preventive practices, suggesting potential gaps in patient education and preventive care integration within healthcare systems.

Finally, country of residence was identified as a significant predictor of preventive behavior, with participants from Syria being less likely to engage in preventive practices compared to those from Saudi Arabia. This finding underscores the critical role of structural and healthcare system factors including access to services, resource availability, and health system capacity—in shaping preventive health behaviors (35). The protracted conflict in Syria over the past decade, along with the resulting socioeconomic challenges, including increased poverty, reduced access to healthcare, and shortages in health services, may further help explain these findings.

### Public health implications

The findings of this study have important implications for public health practice, particularly in enhancing the effective use of family medical history (FMH) for disease prevention and early detection. Although participants demonstrated moderate awareness (53.4%) and predominantly favorable attitudes (74.2%), substantially lower levels of health-related practices (48.5%) and preventive behaviors (44.0%) were observed (Table 3). This discrepancy highlights a critical gap between awareness and actual behavioral engagement, which has been increasingly recognized in recent public health research examining preventive health behavior uptake (43).

These findings suggest that current public health efforts focusing primarily on awareness may be insufficient to drive meaningful behavioral change. Instead, strategies should prioritize facilitating the translation of awareness and positive attitudes into actual preventive practices. In this context, integrating family medical history assessment into routine primary healthcare and digital health systems may improve risk identification and support preventive decision-making (33). Healthcare providers play a key role in this process by actively engaging individuals in discussions about family health history and guiding appropriate preventive actions.

The substantial intention–behavior gap observed in this study indicates that knowledge alone is insufficient to drive action. Behavioral determinants such as perceived susceptibility, perceived barriers, and access to healthcare services are likely influencing individuals’ ability to act on their knowledge (30). Additionally, difficulty in obtaining family medical history information highlights the importance of improving communication and health literacy, as barriers to family-based health information sharing remain a persistent challenge (10).

Notably, significant differences between Saudi Arabia and Syria further emphasize the role of healthcare system factors in shaping preventive behaviors. Variations in access to healthcare services, availability of screening programs, and system-level infrastructure, coupled with socioeconomic challenges and the protracted conflict in Syria, likely contribute to differences in engagement with preventive practices. Similar patterns have been observed in studies examining inequalities in preventive service utilization across different healthcare settings (9, 44).

From a policy perspective, these findings support the development of comprehensive, multi-level interventions that combine education with system-level improvements. Expanding access to affordable screening services, strengthening primary healthcare systems, and integrating preventive services into routine care are essential steps. In addition, community-based and culturally tailored interventions may improve engagement, particularly in populations facing barriers to healthcare access. Evidence suggests that multi-component and context-specific interventions are more effective in improving preventive health behaviors (45).

These findings are also relevant to ongoing preventive health initiatives across the Arab region. In recent years, Ministries of Health in several Arab countries have expanded screening programs, health promotion campaigns, and preventive healthcare services aimed at reducing the burden of chronic and genetic diseases. Strengthening the integration of family medical history assessment within such initiatives may further enhance risk identification, support early detection, and improve the effectiveness of preventive health strategies.

Overall, the study underscores the importance of shifting from awareness-focused approaches toward integrated strategies that address both behavioral and structural determinants of health. Such approaches are essential to bridge the gap between awareness, attitudes, and actual preventive practices, ultimately improving early detection and reducing the burden of genetic and chronic diseases.

### Future research

The findings of this study highlight several important directions for future research. First, given the cross-sectional design, causal relationships between awareness, attitudes, health-related practices, and preventive behaviors could not be established. Longitudinal studies are therefore needed to examine how these domains evolve over time and to better understand the pathways through which awareness and attitudes influence sustained preventive behaviors.

Second, the observed gap between intention and actual engagement in preventive practices warrants further investigation using qualitative and mixed-methods approaches. Future studies should explore underlying behavioral, cultural, and psychological factors that may hinder the translation of positive intentions into action, including perceived risk, health beliefs, and family communication dynamics.

Third, the significant differences identified between Saudi Arabia and Syria suggest the need for country-specific and context-sensitive research. Further studies should examine how healthcare system factors, accessibility of services, and sociocultural influences shape the utilization of family medical history and preventive health behaviors in different settings, particularly in low-resource or conflict-affected environments.

Additionally, intervention-based research is needed to evaluate the effectiveness of strategies aimed at improving the use of family medical history in clinical and community settings. This includes assessing digital health tools, primary care–based interventions, and educational programs designed to enhance risk awareness and promote preventive behaviors.

Finally, future research should consider including more diverse and representative populations, as well as incorporating objective measures of health behavior where possible, to reduce reliance on self-reported data and improve the robustness of findings. Expanding research to include healthcare providers’ perspectives may also provide valuable insights into barriers and facilitators of integrating family medical history into routine care.

Overall, further research is essential to develop and evaluate targeted, evidence-based interventions that can effectively bridge the gap between awareness, attitudes, and preventive health behaviors.

### Strengths and limitations

This study has several notable strengths. First, it adopts a comprehensive and integrated approach by simultaneously assessing family medical history (FMH) awareness, attitudes, health-related practices, and preventive behaviors, allowing for a more holistic understanding of the continuum from perception to action. Second, the comparative design across two countries with differing healthcare systems provides valuable contextual insights into how structural and sociocultural factors influence preventive health behaviors. Third, the relatively large sample size (*n* = 586) enhances the statistical power and supports the reliability of the findings. In addition, the use of a structured questionnaire and standardized data collection procedures enhanced the consistency of data collection, while adherence to the STROBE and CHERRIES guidelines improved the transparency and completeness of study reporting. The inclusion of multivariate regression analysis further allows for the identification of independent predictors across key domains.

Despite these strengths, several limitations should be considered. The cross-sectional design limits the ability to establish causal relationships between awareness, attitudes, practices, and preventive behaviors. Data were collected using a self-administered online questionnaire, which may introduce reporting bias, including recall bias and social desirability bias. Although measures were taken to minimize these effects, such as ensuring anonymity and using standardized questions, they cannot be completely excluded. Additionally, attitudes were assessed using dichotomous (yes/no) response options, which may have limited the ability to capture the full range and intensity of participants’ beliefs compared with Likert-type scales. Although measures were taken to minimize these effects, such as ensuring anonymity and using standardized questions, they cannot be completely excluded.

The use of convenience sampling through online platforms may limit the generalizability of the findings, as participants with internet access and higher digital literacy may be overrepresented. The sample also showed imbalances in certain demographic characteristics, such as gender distribution, which may influence the observed associations. Furthermore, all variables were self-reported, and no objective verification of health behaviors or medical history was possible.

Finally, although the study included participants from two countries, the findings may not be generalizable to other regions with different healthcare systems or sociocultural contexts. Future studies employing longitudinal designs, representative sampling methods, and objective measures of health behavior are recommended to build on these findings and enhance external validity.

## Conclusion

This study provides a comprehensive comparative assessment of family medical history (FMH) awareness, attitudes, health-related practices, and preventive behaviors among participants in Saudi Arabia and Syria. The findings reveal that while awareness was moderate and attitudes toward genetic diseases were largely favorable, engagement in health-related practices and preventive behaviors remained suboptimal. A notable discrepancy was observed between participants’ favorable attitudes and intentions toward preventive actions and their reported preventive behaviors.

Significant differences between the two countries further highlight the influence of healthcare system factors, access to services, and sociocultural contexts on the utilization of family medical history and the adoption of preventive health behaviors. Participants from Saudi Arabia demonstrated higher engagement in screening and preventive practices, whereas those from Syria exhibited lower uptake, reflecting disparities in healthcare accessibility and infrastructure.

Overall, the study underscores the need for integrated public health strategies that move beyond awareness-raising to address both behavioral and structural barriers to preventive care. Improving the use of family medical history, expanding access to screening, and implementing context-specific interventions may help translate awareness into preventive action and support earlier disease detection.

## Data Availability

The raw data supporting the conclusions of this article will be made available by the authors, without undue reservation.
