# Supplementary material for: From awareness to action: a comparative study of family medical history and preventive health behaviors in Saudi Arabia and Syria
Source: Front Public Health. 2026 Jul 1;14:1873367. doi: 10.3389/fpubh.2026.1873367 (PMC13368716; doi:10.3389/fpubh.2026.1873367)
Supplement: Supplementary file 1 [file Data_Sheet_1.PDF]

Figure S1. Diverging bar chart (alternative view of Table 3, optional supplementary)

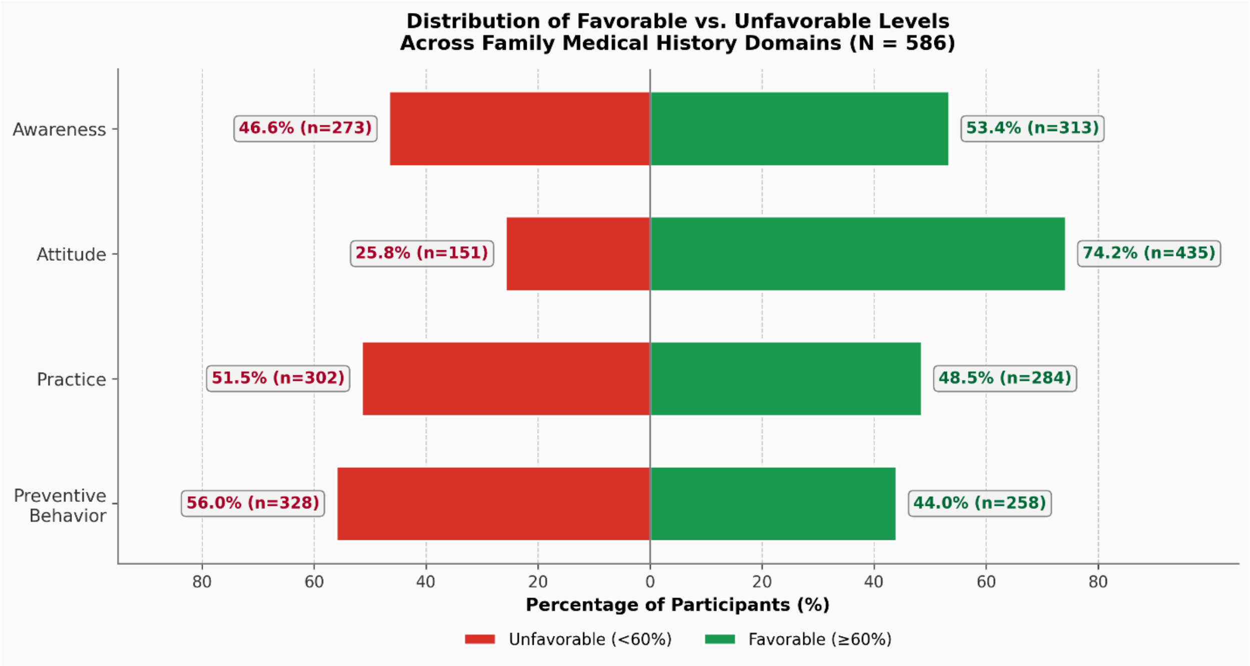

Figure S1. Diverging bar chart (alternative view of Table 3, optional supplementary)
